# Comparison of thymidine phosphorylase expression and prognostic factors in gallbladder and bile duct cancer

**DOI:** 10.1186/1471-2407-10-564

**Published:** 2010-10-19

**Authors:** Hye Sung Won, Myung Ah Lee, Eun-Seon Chung, Dong-Goo Kim, Young Kyoung You, Tae Ho Hong, In-Seok Lee

**Affiliations:** 1 Division of Medical Oncology, Department of Internal Medicine, Uijeongbu St. Mary's Hospital, Kyonggi-do, Korea; 2 Division of Medical Oncology, Department of Internal Medicine, Seoul St. Mary's Hospital, Seoul, Korea; 3 Department of Hospital Pathology, Seoul St. Mary's Hospital, Seoul, Korea; 4 Department of Surgery, Seoul St. Mary's Hospital, Seoul, Korea; 5 Division of Gastroenterology, Department of Internal Medicine, Seoul St. Mary's Hospital, Seoul, Korea

## Abstract

**Background:**

Biliary tract cancers have limitations in information about different location-related pathogenesis and clinico-pathological characteristics. The goal of this study was to investigate anatomical site-related similarities and differences in biliary tract cancers and to assess the expression and clinical significance of functional proteins such as p53, cyclin D1, survivin, thymidine phosphorylase, and ERCC1.

**Methods:**

One hundred and sixty-one patients with biliary tract adenocarcinomas, who underwent curative or palliative surgery in a single institution between October 1994 and December 2003 were evaluated, retrospectively. The level of protein expression of p53, cyclin D1, survivin, thymidine phosphorylase, and ERCC1 was assessed by immunohistochemistry.

**Results:**

With respect to clinico-pathological characteristics, gallbladder cancer was more frequent in women, and bile duct cancer was more common in men. Perineural invasion was more common in bile duct cancer. Recurrence as a distant metastasis was more common in gallbladder cancer. Immunohistochemical analysis revealed that thymidine phosphorylase expression was significantly higher in gallbladder cancer than in bile duct cancer. Positive thymidine phosphorylase and p53 staining were associated with an advanced stage. Differentiation, vascular invasion, perineural invasion, lymphatic invasion, lymph node metastasis, and TNM stage independently predicted poor prognosis in biliary tract cancer. These correlations were seen more clearly in gallbladder cancer. The immunohistochemical staining patterns of p53, cyclin D1, survivin, thymidine phosphorylase, and ERCC1 showed no prognostic significance in biliary tract cancers.

**Conclusions:**

We concluded that gallbladder and bile duct cancers are considered to be separate diseases with different clinico-pathological characteristics and prognostic factors. In addition, we hypothesize that high expression of thymidine phosphorylase by gallbladder cancer results in a higher response rate to capecitabine by gallbladder cancer than bile duct cancer.

## Background

Biliary tract cancers are relatively uncommon cancers in the western countries accounting for approximately 4% of gastrointestinal malignancies, but they are more common in Asia [1]. Based on the data of the National Cancer Registry in 2007, it is the eighth most common cancer in Korea, with an annual incidence of 4,419 cases per 100,000. Although surgical resection remains the only potentially curative treatment, many patients are not eligible for surgery because of the advanced stage at the time of diagnosis. The prognosis for patients with advanced disease is dismal, and the median survival is less than 6 months [1]. Characteristics such as general rarity, difficulty in diagnosis, and overall poor prognosis lead to a paucity of data from which the natural history and optimal treatment regimens can be defined. Additionally, the molecular basis of these tumors and biomarkers related to prognosis or response to chemotherapy remain poorly understood. Biliary tract cancers can be divided into bile duct cancer and gallbladder (GB) cancer. GB and bile duct cancer have been considered similar, and hence have been treated with the same treatment protocols or clinical trials. However, they are currently considered different disease entities, because they have been shown to be distinct in clinical behavior and response to anti-cancer treatments.

5-fluorouracil (5-FU) is the most evaluated drug for GB and bile duct malignancies as a single agent or in combination. Recently, gemcitabine and cisplatin have shown improved survival of patients with biliary tract cancer compared with gemcitabine alone [2]. Thymidine phosphorylase (TP) is an enzyme that converts 5'-deoxy-5-fluorouridine (5'-DFUR) to 5-FU. The expression of TP might be correlated with the efficacy of 5-FU-based chemotherapy [3]. Overexpression of the excision repair cross-complementation group 1 (ERCC1) gene, which is crucial in the repair of cisplatin-induced DNA adducts, has been reported to negatively influence to effectiveness of cisplatin-based chemotherapy [4].

This study aims to compare the clinico-pathological characteristics and prognostic factors of GB and bile duct cancers, and also investigate the expression and clinical significance of TP and ERCC1, including several cell-cycle regulatory proteins in biliary tract cancers.

## Methods

### Patients

One hundred and sixty-one patients who underwent curative or palliative surgery for extrahepatic cholangiocarinoma, Klatskin tumor or GB cancer in Kangnam St. Mary's Hospital between October 1994 and December 2003 were evaluated. We excluded patients with intrahepatic cholangiocarinoma, because this type of cancer has been known to have different clinico-pathological characteristics from the other two types of bile duct cancers. Among 161 patients, 65 (40.4%) patients had been diagnosed with GB cancer, and 96 (59.6%) patients with extrahepatic and Klatskin tumor (BDC). Clinical records and pathological reports were reviewed retrospectively. The following clinical data were collected: patient age, gender, objectives of operation, adjuvant therapy, tumor staging, recurrence, and survival. Ethics committee approval was obtained from the Institutional Review Board of The Catholic University of Korea, Seoul St. Mary's Hospital, Seoul, Korea. Informed consent was provided according to the Declaration of Helsinki.

### Tissue microarray and Immunohistochemical staining

**Figure 1:**
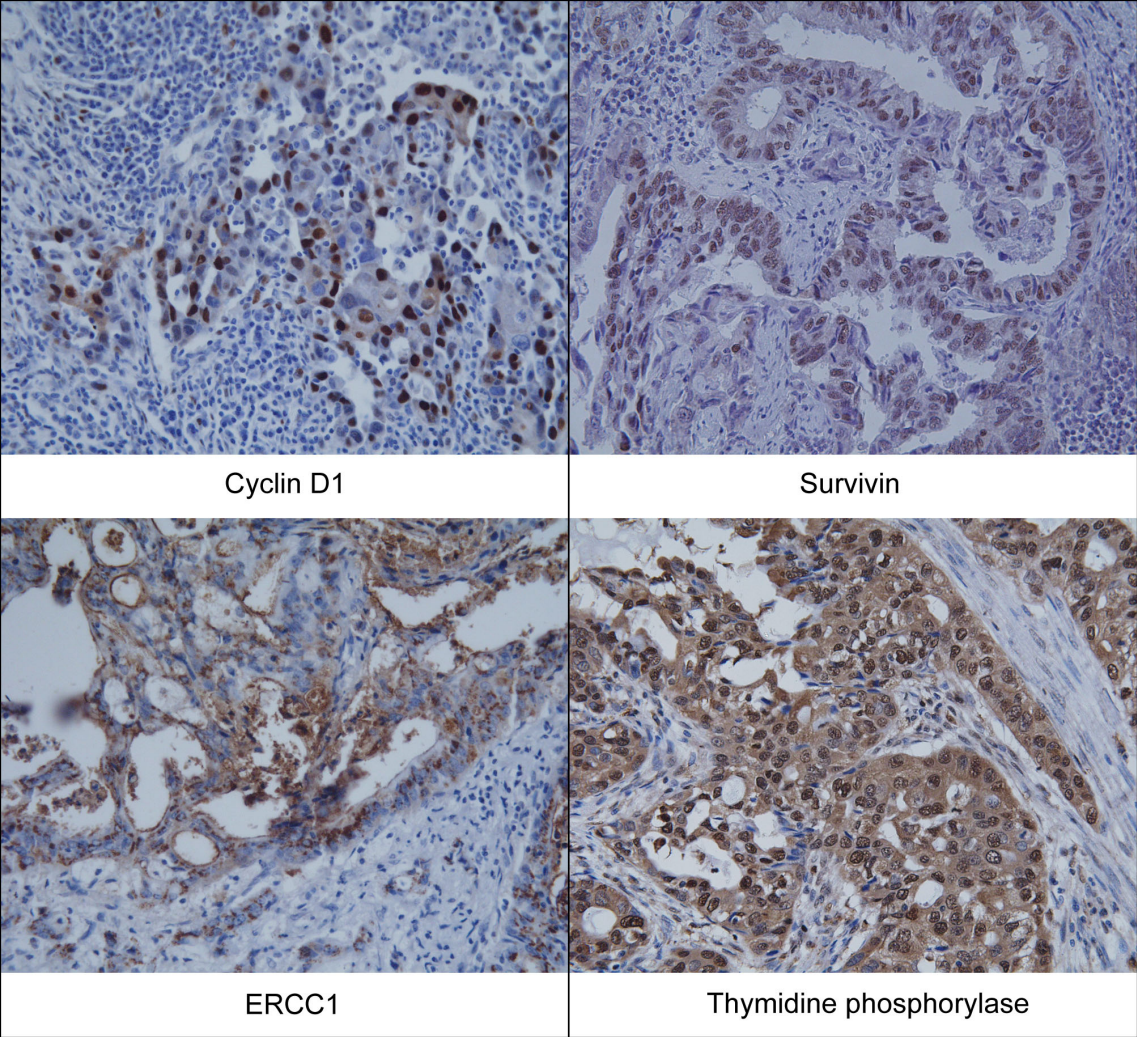
**Cyclin D1, survivin, ERCC1, and TP expressions by immunohistochemistry in biliary tract cancer (magnification ×200)**.

To construct the tissue microarray block, small core biopsies (3.0 mm diameter) were taken from non-necrotic, morphologically representative areas of paraffin-embedded tumor tissues. One hundred and sixty-one tissue cores from each specimen were assembled on a recipient paraffin block with a precision instrument (Micro Digital Co. Korea). After construction, 5 μm sections were cut and hematoxylin-eosin staining was performed on the initial slide for histological verification. Immunohistochemical staining was performed on the 5 μm sections of the tissue microarray blocks. Paraffin sections were deparaffinized in xylene and rehydrated in serial graded ethanol, and then subjected to microwave antigen retrieval. Endogenous peroxidase activity was blocked with 0.3% hydrogen peroxide in methanol. Sections were incubated for 1 hour at room temperature or at 4 ℃ overnight with the following primary antibodies at the specified dilutions: TP (Zymed Laboratories, South San Francisco, CA, USA) diluted 1:100, ERCC1 (NeoMarkers, Fremont, CA, USA) diluted 1:200, survivin (NeoMarkers, Fremont, CA, USA) diluted 1:50, p53 (DAKO Corporation, Carpinteria, CA, USA) diluted 1:100, and cyclin D1 (DAKO Corporation, Carpinteria, CA, USA) diluted 1:50. Immunohistochemical staining was performed using the rabbit or mouse DAKO ChemMate TM EnVision TM system and the Peroxidase/DAB kit (DAKO). Sections were then counterstained with Mayer hematoxylin and dehydrated, cleared and mounted. The results were interpreted by two independent pathologists who were blinded to the specific diagnosis and prognosis for each case. The staining intensity was scored on a three-tiered scale: score 0 = less than 10% of cells positive; 1 = 10-49% positive; and 2 = more than 50% of cells positive. The criterion for positive staining was more than 1 + of tumor cells that showed distinct nuclear or cytoplasmic staining (Figure 1) [5-8].

### Statistical analysis

Overall survival (OS) was defined as the time from the date of diagnosis to the date of death or last follow-up. Time to recurrence (TTR) was defined as the time from the date of diagnosis to the date of recurrence. Continuous and categorical variables were compared using the Student's *t*-test and the chi-square test. Univariate survival analysis and survival curves were estimated using the Kaplan-Meier method, and the log-rank test was applied to assess statistical differences. Multivariate analysis was performed with the Cox hazards regression model. All statistical analyses were performed using the SPSS program (version 15.0) and p < 0.05 was considered statistically significant.

## Results

### Patient characteristics

The clinico-pathological characteristics of the 161 patients included in our study are summarized in Table 1. The median age of the patients was 62 (range 35-87) years. GB cancer was more frequent in women, and BDC was more common in men (p = 0.001). There was no significant difference in age, stage and resectability between GB cancer and BDC. Among 161 patients, 127 patients experienced surgical resection with curative intent, and 70 patients (55.1%) took adjuvant therapy with chemotherapy or chemo-radiation based on 5-FU.

**Table 1:** Clinico-pathological characteristics of the biliary tract cancer patients

Characteristics	All patients (n = 161)		GB cancer (n = 65)		Bile duct cancer (n = 96)		p-value
				
	No. of patients	%	No. of patients	%	No. of patients	%	
Age, years							0.265
Mean ± SD	61.9 ± 9.8		63.4 ± 11.4		60.9 ± 8.5		
Sex							**0.001**
Male	95	59.0	28	43.1	67	69.8	
Female	66	41.0	37	56.9	29	30.2	
Resectability							0.242
Curative	127	78.9	49	75.4	78	81.3	
Palliative	34	21.1	16	24.6	18	18.7	
Adjuvant therapy	70	43.4	30	46.1	40	41.6	0.229
Histopathology							
Well differentiated	51	31.6	23	35.3	28	29.1	0.243
Vascular invasion	13	8.0	5	7.6	8	8.3	0.606
Perineural invasion	82	50.9	15	23.0	67	69.7	**0.001**
Lymphatic invasion	53	32.9	24	36.9	29	30.2	0.161
LN metastases	60	37.2	25	38.4	35	36.4	0.381
TNM stage							0.105
I	56	35.0	22	33.8	34	35.8	
II	66	41.3	24	36.9	42	44.2	
III	14	8.7	4	6.2	10	10.5	
IV	24	15.0	15	23.1	9	9.5	

GB, gallbladder; SD, standard deviation; LN, lymph node.

### Immunohistochemical staining and correlation with clinico-pathological variables

Histopathology showed that there was no significant difference in differentiation, vascular invasion, and lymphatic invasion between GB cancer and BDC. However, BDC showed significantly higher perineural invasion compared to GB cancer (p = 0.001) (Table 1). The immunohistochemical staining results are listed in Table 2. Immunohistochemistry results of some tissue cores cannot be determined, because of inadequate or incorrect expression and loss of tissue samples in the tissue microarray. Among all cases on which interpretation of immunohistochemistry was possible, 72 cases (56.7%) and 35 cases (26.7%) showed positive expression for p53 and cyclin D1, respectively, 54 cases (41.9%) for survivin, and 78 cases (65.0%) for TP. However, ERCC1 expression was mostly negative in 74 cases (65.5%). In comparison, TP expression was significantly higher in GB cancer than in BDC (p = 0.006). We analyzed the correlations between the clinico-pathololgical findings and the expression of these proteins (Table 3). For all tumors, positive TP and p53 staining was associated with an advanced stage compared to tumors with negative staining (p = 0.027 and p = 0.009, respectively). Other significant correlations with clinico-pathological variables were not observed.

**Table 2:** Immunohistochemical staining for tumors stratified by anatomical sites

Variable	All patients		GB cancer		Bile duct cancer		p-value
				
	**No**.	%	**No**.	%	**No**.	%	
P53 (n = 127)							
Positive	72	56.7	32	61.5	40	53.3	0.231
Negative	55	43.3	20	38.5	35	46.7	
Cyclin D1 (n = 131)							
Positive	35	26.7	18	33.3	17	22.1	0.109
Negative	96	73.3	36	66.7	60	77.9	
Survivin (n = 129)							
Positive	54	41.9	20	37.7	34	44.7	0.271
Negative	75	58.1	33	62.3	42	55.3	
TP (n = 120)							
Positive	78	65.0	40	78.4	38	55.1	**0.006**
Negative	42	35.0	11	21.6	31	44.9	
ERCC1 (n = 113)							
Positive	39	34.5	18	36.7	21	32.8	0.406
Negative	74	65.5	31	63.3	43	67.2	

GB, gallbladder; TP, thymidine phosphorylase; ERCC1, excision repair cross complementing protein 1

**Table 3:** Immunohistochemical staining for tumors stratified by clinico-pathological variables

Variable	TP		ERCC1		Cyclin D1		Survivin		P53	
	
	**No**.	p	**No**.	p	**No**.	p	**No**.	p	**No**.	p
Differentiation										
Well	18	0.096	11	0.557	9	0.405	14	0.344	17	0.190
Others	57		28		26		39		51	
Vascular invasion										
Yes	10	0.112	3	0.383	3	0.592	4	0.495	6	0.459
No	61		32		30		46		60	
Perineural invasion										
Yes	42	0.171	23	0.087	19	0.528	30	0.258	40	0.222
No	28		11		14		19		25	
Lymphatic invasion										
Yes	30	0.350	17	0.181	14	0.311	16	0.170	28	0.226
No	41		18		19		34		38	
LN metastasis										
Yes	32	0.310	19	0.126	14	0.470	24	0.091	18	0.274
No	42		19		21		29		31	
T stage										
1	7	**0.027**	2	0.083	3	0.442	10	0.806	8	**0.009**
2	22		14		10		12		16	
3	35		15		17		26		41	
4	14		8		5		6		7	

TP, thymidine phosphorylase; ERCC1, excision repair cross complementing protein 1

### Clinical outcome: recurrence & survival analysis

**Figure 2:**
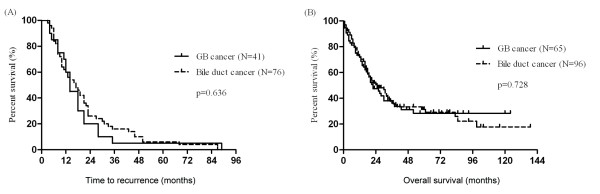
**Kaplan-Meier survival curves for time to recurrence (A) and overall survival (B) in biliary tract cancer according to anatomical sites**.

Among 127 patients with curative resection, the assessment of recurrence was possible in 117 patients. Seventy patients (59.8%) developed recurrence at a median of 14 months. Recurrence occurred in 50 patients (65.7%) with BDC and 20 patients (48.7%) with GB cancer (p = 0.049). The median TTR for patients with GB cancer and BDC were 14 and 17 months, respectively (p = 0.636) (Figure 2A). The patterns of recurrence were classified into three types; local, regional, and distant recurrence. Local recurrence is defined as recurrence of the cancer at the resection sites. Regional recurrence is defined as recurrence at the lymph nodes near the primary site. Distant recurrence is defined as the recurrence of the cancer in an organ distant from the primary site. Local recurrence was more common in BDC (32.0% vs 5.0%), while recurrence as a distant metastasis was more common in GB cancer (35.0% vs 18.0%) (p = 0.041). The correlation analysis with protein expression showed that there was no significant association of protein expression with recurrence rate, pattern, or TTR.

The median OS time of patients with GB cancer and BDC was 20 and 21 months, respectively. There was no significant difference in OS (p = 0.728) (Figure 2B). In univariate analysis, the significant factors affecting OS were differentiation, vascular invasion, perineural invasion, lymphatic invasion, lymph node metastasis, and TNM stage (Table 4). In a subgroup analysis stratified by type of cancer, these correlations were observed to be identical in GB cancer. Meanwhile, only two factors, differentiation and perineural invasion, were significantly correlated with survival time in BDC. In multivariate survival analysis, differentiation and TNM stage showed a significant impact on OS in GB cancer (HR = 0.472, 1.532; p = 0.039, 0.042). Meanwhile, in BDC, TNM stage was only significant prognostic factor affecting survival (HR = 2.312; p = 0.001). The expression of p53, cyclin D1, survivin, TP, and ERCC1 did not independently predict poor outcome in univariate analysis. Among the 66 patients who received adjuvant treatment, there was no significant difference in TTR and OS associated with TP and ERCC1 expression (p = 0.212, 0.322 for TP; p = 0.591, 0.794 for ERCC1).

**Table 4:** Univariate survival analysis according to clinico-pathological variables

Variable		All patients		GB cancer		Bile duct cancer	
		
		Median Survival	p	Median Survival	p	Median Survival	p
Anatomic location	GB	22	0.939				
	Bile duct	24					
Well differentiated	Yes	99	**0.001**	.	**0.001**	85	**0.001**
	No	18		17		19	
Vascular invasion	Positive	13	**0.022**	12	**0.029**	13	0.261
	Negative	27		27		26	
Perineural invasion	Positive	19	**0.001**	16	**0.029**	19	**0.005**
	Negative	40		30		.	
Lymphatic invasion	Positive	18	**0.011**	16	**0.047**	18	0.080
	Negative	31		30		31	
Lymph node metastasis	Positive	18	**0.001**	38	**0.001**	21	0.058
	Negative	32		10		30	
Stage	1	83	**0.001**	.	**0.001**	36	0.069
	2	22		20		26	
	3	12		9		13	
	4	5		5		8	
P53	Positive	20	0.210	19	0.409	24	0.374
	Negative	30		30		35	
Cyclin D1	Positve	25	0.890	20	0.943	25	0.962
	Negative	22		21		32	
Survivin	Positive	27	0.339	20	0.995	58	0.267
	Negative	22		21		25	
TP	Positive	22	0.463	22	0.625	22	0.194
	Negative	21		19		32	
ERCC1	Positive	18	0.241	21	0.903	14	0.073
	Negative	27		20		33	

GB, gallbladder; TP, thymidine phosphorylase; ERCC1, excision repair cross complementing protein 1

## Discussion

Biliary tract cancer is a heterogeneous disease with diverse anatomical sites. In the present study, we investigated the entire spectrum of biliary tract cancer to define anatomical site-related similarities and differences. Several differences were observed between GB cancer and BDC. In clinico-pathological characteristics, GB cancer was more frequent in women, and BDC was more common in men. These sex differences are probably related to the higher incidence of gallstones in women and of primary sclerosing cholangitis in men, which are known risk factors for GB cancer and BDC, respectively [1]. Another anatomical site-related difference was the frequency of perineural invasion. Perineural invasion is a distinct pathological entity that can be observed in the absence of lymphatic and vascular invasion [9]. Although the pathogenesis and clinical significance of perineural invasion remain unclear, it is considered an under-recognized route of metastatic spread. For some malignancies including head and neck, pancreas, colorectum, and prostate, perineural invasion has been associated with a lower 5 year survival rate and a more advanced disease stage [9]. In our study, perineural invasion was observed more frequently in BDC than in GB cancer. Additionally, univariate analysis revealed that perineural invasion was one of the prognostic factors in biliary tract cancer. Meanwhile, some differences were found in recurrence patterns and prognostic factors between GB cancer and BDC. BDC had higher local recurrence than GB cancer, while recurrence as a distant metastasis was more common in GB cancer. These differences should be considered to determine adjuvant therapy after curative resection. In univariate analysis, differentiation, vascular invasion, perineural invasion, lymphatic invasion, lymph node metastasis, and TNM stage independently predicted poor prognosis in biliary tract cancer. These correlations were seen more clearly in GB cancer than in BDC. Also, in multivariate analysis, differentiation and TNM stage showed a prognostic impact on survival in biliary tract cancer. Taken together, GB cancer and BDC are thought to be separate diseases with differences in pathogenesis influencing clinical behavior.

We assessed the expression of p53, cyclin D1 and survivin, to compare the differences in molecular changes associated with the prognosis between BDC and GB cancer. Apoptosis and cell cycle control are processes required for the regulation of cellular homeostasis. Chronic imbalance between apoptosis and cell proliferation is the first step of carcinogenesis in all tumors. Therefore, prognostic significance of cell cycle regulatory protein such as p53 and cyclin D1 and anti-apoptosis protein, survivin, has been investigated in malignancies [10-13]. Kim et al. reported the expression and clinical significance of cell cycle regulatory proteins in biliary tract cancer. They presented that cyclin D1 overexpression was more common in extrahepatic bile duct cancer than in GB cancer. In univariate analysis, cyclin D1 and p53 showed no prognostic impact on survival in biliary tract cancer [10]. Jarnagin et al. also presented that biliary tract cancers differentially expressed cell cycle regulatory protein based on tumor location and morphology. According to their results, cyclin D1 overexpression varied based on anatomic site, and it was less common in GB cancer. However, there was no difference in p53 overexpression according to anatomic site, and prognostic roles were not identified for cyclin D1 and p53 [11]. In our study, there was no difference in p53 and cyclin D1 overexpression based on anatomic site, and also they were no prognostic impact on survival in BDC and GB cancer, respectively. Survivin is a member of the inhibitor of apoptosis family that regulates cell division and suppresses apoptosis [8]. In previous reports, survivin overexpression was correlated with poor prognosis in some gastrointestinal malignancies such as gastric cancer and pancreatic cancer [8]. Javel et al. also reported that nuclear survivin expression was associated with poor prognosis in cholangiocarcinoma [14]. However, in our study, there was no difference in survivin expression according to anatomic site, and no correlation between survivin expression and prognosis in biliary tract cancer.

Additionally, we examined the expression of TP and ERCC1 associated with the response to 5-FU and cisplatin chemotherapy [15]. No significant differences were found in the expression of p53, cyclin D1, survivin, and ERCC1 between GB cancer and BDC. However, TP expression was significantly higher in GB cancer than in BDC. This result can explain why these two cancers have shown different response rates to capecitabine combination chemotherapy in many clinical trials. TP represents the rate-limiting enzyme in the activation of 5'-DFUR and capecitabine. The 5-FU prodrug capecitabine is metabolized to 5-FU through a three-step enzymatic conversion. In the final step, 5'-DFUR is metabolized to the active form 5-FU by TP. Therefore, the sensitivity of tumor cells to capecitabine might be enhanced by increasing TP expression [3,16]. Actually, Kim et al. presented results from a phase II study of capecitabine plus cisplatin as a first-line chemotherapy for advanced biliary cancer. According to their results, GB cancer appeared to respond better to capecitabine and cisplatin treatment than BDC (32% vs 13%) [17,18]. In another phase II study with capecitabine and oxaliplatin, Nehls et al. reported that the response rates for GB, extrahepatic, and intrahepatic cholangiocarcinoma were 30%, 25%, and 0%, respectively [19]. In this study, we cannot access the clinical correlations between TP expression and response to capecitabine and survival, because capecitabine for biliary tract cancers was unavailable in Korea during 1994-2003. However, based on our results, correlative studies will be warranted. Also, in the future, randomized trials of biliary tract cancer should be performed based on stratification by anatomical site. Meanwhile, correlation studies with clinico-pathological variables revealed a significant association between positive TP and p53 staining with an advanced stage of the disease. These results are in accordance with previous studies, and the pro-angiogenic activity of TP can explain the more aggressive behavior of TP-expressing tumors [3].

## Conclusions

In summary, there were some differences in clinico-pathological characteristics, prognostic factors, and recurrence patterns between GB cancer and BDC. Therefore, these differences should be considered when planning clinical trials and treatments. Additionally, further studies may be required to evaluate whether TP expression can be used as a predictive marker for the clinical response to capecitabine chemotherapy in GB cancer.

## Competing interests

The authors declare that they have no competing interests.

## Authors' contributions

HSW drafted the manuscript. HSW, MAL, DGK, YKY, THH and ISL collected the data and followed the patients. ESC evaluated H&E stained and immunostained slides. DGK, YKY, and THH provided the clinical specimens for this study and clinical perspective. MAL designed the study and helped with the manuscript. All authors read and approved the final manuscript.

## Pre-publication history

The pre-publication history for this paper can be accessed here:

http://www.biomedcentral.com/1471-2407/10/564/prepub
